# Psychometric properties of the reduced version of the Primary Care Assessment Tool (PCATool)

**DOI:** 10.1590/1980-549720240057

**Published:** 2024-12-09

**Authors:** Gabriele Rissotto Menegazzo, Maria Laura Braccini Fagundes, Orlando Luiz do Amaral, Lucelen Fontoura Bastos, Amanda Ramos da Cunha, Matheus Neves, Eduardo Dickie de Castilhos, Otávio Pereira D'Avila, Jessye Melgarejo do Amaral Giordani, Fernando Neves Hugo

**Affiliations:** I Universidade Federal de Santa Maria, Postgraduate Program in Dental Sciences – Santa Maria (RS), Brazil.; II Universidade Federal do Rio Grande do Sul, Postgraduate Program in Dentistry – Porto Alegre (RS), Brazil.; III Universidade de São Paulo, Department of Epidemiology – São Paulo (SP), Brazil.; IV Universidade Federal de Pelotas, Department of Preventive and Social Dentistry – Pelotas (RS), Brazil.

**Keywords:** Primary health care, Health services research, Health surveys, Validation study, Atenção primária à saúde, Pesquisa sobre serviços de saúde, Inquéritos epidemiológicos, Estudo de validação

## Abstract

**Objective:**

This cross-sectional study aimed to evaluate the psychometric properties of the reduced version of the Primary Care Assessment Tool (PCATool) for adult patients in the Brazilian National Health Survey 2019, a nationally representative population-based study.

**Methods:**

The reduced version of PCATool-adults measures the presence and extent of the following attributes: degree of affiliation; first-contact access; longitudinality; care coordination; comprehensiveness; family orientation; and community orientation. Exploratory and Confirmatory Factor Analysis were performed.

**Results:**

The final sample consisted of 9,396 individuals. The PCATool latent variable for adults was related to two questionnaire items, one referring to the Degree of Affiliation attribute and the other to the Community Orientation attribute. Additionally, five latent variables, formed by 21 items, identified the other five attributes through standardized factor loadings.

**Conclusion:**

The reduced version of PCATool-Adult has good psychometric properties and captures the attributes of Primary Health Care.

## RESUMO

**Objetivo:**

Este estudo transversal teve como objetivo avaliar as propriedades psicométricas da versão reduzida do *Primary Care Assessment Tool* (PCATool), versão para pacientes adultos, na Pesquisa Nacional de Saúde de 2019, um estudo populacional de representatividade nacional.

**Métodos:**

A versão reduzida do PCATool para adultos mede a presença e a extensão dos seguintes atributos: grau de afiliação; acesso ao primeiro contato; longitudinalidade; coordenação do cuidado; integralidade; orientação familiar; e orientação comunitária. Foram realizadas Análise Fatorial Exploratória e Análise Fatorial Confirmatória.

**Resultados:**

A amostra final consistiu em 9.396 pessoas. A variável latente do PCATool para adultos foi relacionada a dois itens do questionário, um referente ao atributo de Grau de Afiliação e outro ao atributo de Orientação Comunitária, além de cinco variáveis latentes formadas por 21 itens, que demonstraram os outros cinco atributos, por meio de de cargas fatoriais padronizadas.

**Conclusão:**

A versão reduzida do PCATool para adultos possui boas propriedades psicométricas e captura os atributos da Atenção Primária à Saúde.

## INTRODUCTION

The guiding principles of Primary Health Care (PHC) have been outlined in the declaration of Alma-Ata. PHC aims to address the majority of a person's health needs throughout life^1^. Mendes^2^ identified three main interpretations of PHC;

1. Selective (understood as a specific program intended for poor populations and regions, offering a limited set of simple, low-cost healthcare interventions that are generally provided by mid-level health care workers and without the possibility of referral to higher levels of technological care;
2. PHC as the first level of a health system; and
3. PHC as a strategy to organize a health system, designed to produce sustainable health improvements and greater health equity^1,2^.

PHC has several structuring attributes essential for its effectiveness: first contact access, longitudinality, comprehensiveness, and care coordination; it also has derived attributes that qualify the actions of PHC: family orientation, community orientation, and cultural competence. These seven attributes refer to the degree to which people seek out PHC, the degree of connection and relationship between PHC and people under its care, the resolution capacity, and the ability to coordinate cases and care flows^1,3^. In Brazil, the Ministry of Health has been developing strategies to strengthen PHC, including the systematic assessment of the quality of PHC services over the past three decades^3,4^.

The evaluation of health services is based on measuring aspects related to structure, processes, and outcomes, building on the work by Avedis Donabedian on healthcare quality, initiated in the 1960s. Structure encompasses the human, physical, and financial resources used to provide healthcare, as well as the organizational parameters and means of financing these resources. Process encompasses the constitutive actions of healthcare, including the organization of the work process in the provision of health services. Outcomes refer to changes in the population's health conditions promoted by the care received^5^. The Primary Care Assessment Tool (PCATool) was developed to assess the degree of orientation toward PHC in health services organized under different models. The degree of orientation toward PHC refers to how well PHC services align with the principles and practices of PHC, including accessibility, comprehensiveness, coordination, continuity, and community orientation^3^.

The development and validation of PCATool were initially carried out in the United States of America (USA), resulting in a set of 92 related items with sufficient reliability and validity^6^. Different versions of PCATool were later developed in Brazilian Portuguese (*e.g*., adult patients, infant patients, nurses & physicians, oral health), confirming its adequacy for evaluating PHC services, but also evidencing the small contribution of some items and suggesting the possibility of reduced versions, better suited to large-scale surveys^7^. Therefore, a reduced version of the PCATool-adults was developed using Item Response Theory in a Brazilian sample. The findings of this study showed that a reduced version of PCATool could adequately evaluate PHC services, however prior research on the psychometric properties of PCATool-Adults relied solely on exploratory factor analysis^8^.

Considering that:

1. The evaluation of PHC is relevant to support decision-making and to verify the effectiveness of healthcare provision;
2. The need for reduced instruments more suitable for large-scale surveys;
3. The last analysis of the psychometric properties of the PCATool was carried out over a decade ago; and
4. The Brazil National Health Survey 2019 (*Pesquisa Nacional de Saúde* 2019) represents an opportunity to resume and strengthen the analysis of these properties with a robust, nationally representative sample, the aim of this study was to evaluate the psychometric properties of the reduced version of Primary Care Assessment Tool for adult patients in PNS 2019.

## METHODS

PNS 2019 was approved by the National Research Ethics Commission (3.529.376), and prior written informed consent was obtained from all participants. The PNS data are available online for public access and use at the Brazilian Institute of Geography and Statistics (*Instituto Brasileiro de Geografia e Estatística* – IBGE) official website (https://www.ibge.gov.br/estatisticas/sociais/saude/9160-pesquisa-nacional-de-saude).

### Study design and sample

This cross-sectional study analyzed data from the second edition of a nationally representative population-based study. The first edition was carried out in 2013, and the second in 2019. Interviews were carried out between August 2019 and March 2020. The PNS sample originated from a master sample, consisting of a set of units from selected areas in a register, designed to meet the sampling requirements of several surveys included in the Integrated Home Survey System (IHS/IBGE), such as the National Household Sample Survey and the Household Budget Survey. The target population comprised individuals aged 15 years old or older, living in permanent private households. PNS employed a cluster sampling plan with three stages: the census tracts in the Primary Sampling Units were the first stage; the household was the second stage; and within each household, a person aged 15 years old or older (randomly selected) constituted the third stage. The PCATool-adults version is aimed at individuals aged 18 and over, therefore, the present study included data from participants aged 18 years old or older. In PNS 2019, a total of 94,114 individuals were interviewed. Of these, 88,531 participants aged 18 years old or older were selected for the sample. Since only individuals who reported seeking care at a primary healthcare facility (such as a health post, health center, or Family Health Unit) during their last medical visit answered the PCATool, the final sample consisted of 9,396 people. Additional information on sampling, including the methods for defining sample size, can be found in a specific publication about PNS 2019^9^.

### Data collection

Data were obtained through structured interviews carried out by trained interviewers at the participants’ homes. A household questionnaire and an individual questionnaire were used.

PCATool Brazil, in its reduced version, was used for data collection. This instrument contains 25 questions, as shown in Table 1, and measures the presence and extent of the following attributes: affiliation; first contact access; longitudinality; care coordination; comprehensiveness; family orientation; and community orientation. The extent refers to the degree or intensity to which each attribute is present and implemented in the evaluated health services.

**Table 1:** Questions from the reduced version of the Primary Care Assessment Tool according to their attributes.

PCATool Attribute	Item	Question	Portuguese version
Degree of Affiliation	A1	Is there a health service/doctor/nurse you usually go to when you get sick or need advice about your health?	Há um(a) serviço de saúde/médico(a)/enfermeiro(a) onde você geralmente vai quando adoece ou precisa de conselhos sobre a sua saúde?
	A2	Is there a health service/doctor/nurse who knows you best as a person?	Há um(a) serviço de saúde/médico(a)/enfermeiro(a) que melhor conhece você como pessoa?
	A3	Is there a health service/doctor/nurse who is most responsible for your health care?	Há um(a) serviço de saúde/médico(a)/enfermeiro(a) que é mais responsável por seu atendimento de saúde?
First Contact	B2	When you have a new health problem, do you go to the "health facility/doctor/nurse" before going to another health facility?	Quando você tem um novo problema de saúde, você vai ao(à) "serviço de saúde/médico(a)/enfermeiro(a)" antes de ir a outro serviço de saúde?
	C4	When the "health service" is open, can you get quick advice over the phone or through a virtual communication tool (*e.g.* whatsapp, telegram, wechat, skype, hangout, email) if you need it?	Quando o(a) "serviço de saúde" está aberto(a), você consegue aconselhamento rápido pelo telefone ou por ferramenta de comunicação virtual (ex.: *whatsapp, telegram, wechat, skype, hangout, e-mail*) se precisar?
	C11	Is it difficult for you to get medical care at the "health service" when you think it is needed?	É difícil para você conseguir atendimento médico no(a) "serviço de saúde" quando pensa que é necessário?
Longitudinality	D1	When you go to the "health service," is it the same doctor or nurse who sees you every time?	Quando você vai ao(à) "serviço de saúde", é o(a) mesmo(a) médico(a) ou enfermeiro(a) que atende você todas às vezes?
	D6	Do you feel comfortable sharing your concerns or problems with the "doctor/nurse?"	Você se sente à vontade contando as suas preocupações ou problemas ao(à) "médico(a)/enfermeiro(a)"?
	D9	Does the "doctor/nurse" know which problems are most important to you and your family?	O(a) "médico(a)/enfermeiro(a)" sabe quais problemas são mais importantes para você e a sua família?
	D14	If it were too easy, would you switch from the "health service" to another health service?	Se fosse muito fácil, você mudaria do(a) "serviço de saúde" para outro serviço de saúde?
Care Coordination	E2	Did the "doctor/nurse" suggest (indicate, refer) you to consult with this specialist or in the specialized service?	O(a) "médico(a)/enfermeiro(a)" sugeriu (indicou, encaminhou) que você fosse consultar com esse(a) especialista ou no serviço especializado?
	E6	Did the "doctor/nurse" send any information to the specialist about the reason for this consultation (with the specialist or in the specialized service)?	O(a) "médico(a)/enfermeiro(a)" enviou alguma informação para o(a) especialista sobre o motivo dessa consulta (com o(a) especialista ou no serviço especializado)?
	E7	Does the "doctor/nurse" know the results of the consultation with the specialist or in the specialized service?	O(a) "médico(a)/enfermeiro(a)" sabe quais foram os resultados da consulta com o(a) especialista ou no serviço especializado?
	E9	Did the "doctor/nurse" seem interested in the quality of care you received in the consultation with the specialist or in the specialized service (asked if you were well or poorly treated)?	O(a) "médico(a)/enfermeiro(a)" pareceu interessado(a) na qualidade do cuidado que você recebeu na consulta com o(a) especialista ou no serviço especializado (perguntou se você foi bem ou mal atendido)?
	F3	If you wanted, could you read (consult) your chart at/with the "health service/doctor/nurse?"	Se quisesse, você poderia ler (consultar) o seu prontuário no(a)/com o(a) "serviço de saúde/médico(a)/enfermeiro(a)"?
Comprehensiveness		Indicate whether in the "health service" these options are available (they can be found/obtained):	Indique se no(a) "serviço de saúde" essas opções estão disponíveis (podem ser encontradas/obtidas):
	G9	Counseling for mental health issues (*e.g.*, anxiety, depression)	Aconselhamento para problemas de saúde mental (ex.: ansiedade, depressão)
	G17	Smoking advice (*e.g.*, how to quit smoking)	Aconselhamento sobre tabagismo (ex.: como parar de fumar)
	G20	Counseling about changes that happen with aging (*e.g.*, memory impairment, risk of falling)	Aconselhamento sobre as mudanças que acontecem com o envelhecimento (ex.: diminuição da memória, risco de cair)
		Please, respond if the following topics have already been or are discussed (talked) with you?	Por favor, responda se os seguintes assuntos já foram ou são discutidos (conversados) com você?
	H1	Guidelines on healthy eating, good hygiene and adequate sleep (sleep enough)	Orientações sobre alimentação saudável, boa higiene e sono adequado (dormir suficientemente)
	H5	Guidance on exercise that is right for you	Orientações sobre exercícios físicos apropriados para você
	H7	Check and discuss the medications you are using	Verificar e discutir os medicamentos que você está usando
	H11	How to prevent falls	Como prevenir quedas
Family Orientation	I1	Does the "doctor/nurse" ask your ideas and opinions (what you think) when planning treatment and care for you or someone in your family?	O(a) "médico(a)/enfermeiro(a)" pergunta as suas ideias e opiniões (o que você pensa) ao planejar o tratamento e cuidado para você ou para alguém da sua família?
	I3	Did the "doctor/nurse" meet with members of your family if you felt it necessary?	O(a) "médico(a)/enfermeiro(a)" se reuniria com membros de sua família se você achasse necessário?
Community Orientation	J4	Please indicate if the "health service" performs this initiative. Patient surveys to see if services are meeting people's needs.	Pesquisas com os pacientes para ver se os serviços estão satisfazendo (atendendo) as necessidades das pessoas.

PCATool: Primary Care Assessment Tool.

Responses, except for the affiliation questions, were given on a 4-point Likert scale, as follows: 4 for "definitely yes;" 3 for "probably yes;" 2 for "probably not;" and 1 for "certainly not." In addition, code 9 is assigned when participants "do not know" or "do not remember."

According to the PCATool manual^3^, it was necessary to reverse the answers for items C11 and D14, as these items were formulated in such a way that higher values on the response scale suggested lack/absence of the characteristics being measured in the services. For score calculation, however, higher values on the scale should reflect the presence of these characteristics in the services. Therefore, it is necessary to invert the scale of C11 and D14 as follows: (4=1), (3=2), (2=3), and (1=4). In addition, when the sum of items with missing values or 9 codes were equivalent to 50% or more of the items, the participants were excluded from the analysis. When less than 50% of the items had missing values or 9 codes, values were imputed as 2 ("probably not"), in line with the PCATool Manual, when there were characteristics that were not recognized by the interviewee. Higher scores imply better performance in each attribute, *i.e*., the health service is more PHC-oriented. Also, the affiliation attribute was calculated through items A1, A2, and A3, with a score of 1 in case all answers were "no," a score of 2 in case one answer was "yes," a score of 3 in case of two answers being "yes," and score 4 if all answers wee "yes"^3^. To calculate the overall PHC score, the affiliation score was added to the other items and then divided by 23, the total number of items^3^.

### Statistical analysis

This section outlines the methods employed to assess the degree of orientation of services toward PHC using the PCATool in the Brazilian context. Data were analyzed using the software STATA 14.0 (Stata Corporation, College Station, TX, USA) and Mplus 6.12 (Muthén & Muthén, Los Angeles, CA, USA). A descriptive analysis was performed on the means and standard deviations of the PCATool scores. To standardize the scale, the original scores were converted to a range from zero to 10. This process involved three steps: first, the minimum value of the scale was subtracted from each score. Next, the resulting value was divided by the range of the scale, which is the difference between the maximum and minimum values. Finally, the result was multiplied by 10 to fit the new scale. This procedure, which is well-documented in previous literature^3^, standardized the scores, making the results easier to interpret. Subsequently, Exploratory Factor Analysis (EFA) and Confirmatory Factor Analysis (CFA) were performed to assess the structure of correlations between the items and confirm the items that make up each attribute (*e.g*., constructs) of the PCATool-adults version. All analyses were performed considering the sample weight provided by IBGE, due to the complex sampling.

EFA verified whether the PCATool behaves as a single latent variable or splits according to its seven attributes. Hypothesis tests were conducted to determine the factor matrix that provided the best fit, characterized by the highest factor loadings and the lowest residual error (variance). Specifically, Bartlett's test of sphericity and the Kaiser-Meyer-Olkin (KMO) test were used to evaluate the adequacy of the correlation matrix. These tests ensured that the final version of the correlation matrix was suitable by comparing it to a reference matrix. Bartlett's sphericity test values with significance levels lower than 0.05 indicate that the matrix is factorable, rejecting the null hypothesis that the matrix is similar to the identity matrix. The KMO test measures sampling adequacy by comparing the observed correlations among variables with their partial correlations. A KMO value greater than 0.70 suggests that the variables have sufficient common variance, making them suitable for factor analysis^10^. Essentially, a higher KMO value indicates that the observed correlations are strong enough compared to the partial correlations, ensuring that the data is appropriate for uncovering underlying factor structures. The number of factors to be extracted was determined based on the global fit indices of the measurement model. Additionally, factor rotation, a critical step in PCA, was assessed to improve clarity and interpretability of the results, allowing for a better understanding of the underlying data patterns. The Maximum Likelihood estimator for complex samples (MLR) was employed with robust standard errors^11^.

The PCATool-adults latent variable, then, was formed through theoretical and statistical coherence, and its dimensional structure was confirmed through the evaluation of both individual and global parameters. The performance of the items was analyzed through their respective loadings and residuals. To assess the overall fit of the model, several indices were analyzed: Comparative Fit Index (CFI), Tucker-Lewis Index (TLI), Standardized Root Mean Square Residual (SRMR), and Root Mean Square Error of Approximation (RMSEA), along with its 90% Confidence Intervals (90%CI). For a model to be considered a good fit, CFI and TLI should be greater than or equal to 0.90, RMSEA should be less than or equal to 0.05, and SRMR should be less than or equal to 0.08. These criteria help determine whether the model adequately represents the data^12^. Finally, the general consistency of the best-fitted model was tested using Cronbach's Alpha, which is considered acceptable when values are above 0.7^13^.

## RESULTS

The final sample consisted of 9,396 individuals aged 18 years old or older who responded to the short version of PCATool-adults. Table 2 presents the descriptive statistics for both the overall PCATool-adults scores and its individual attributes. The mean overall score was 5.61 (standard deviation [SD]: 1.77). The attribute with the highest mean was Longitudinality, with 6.75 (SD: 2.48) while the lowest mean was Community Orientation, with 4.10 (SD: 3.94). To conduct EFA, the 25 questions from the reduced Brazilian adult version of the PCATool were tested, considering 1 to 7 factors. The analysis revealed that the data behaved as a latent variable divided into seven distinct domains, determined by evaluating the highest factor loadings, lowest residual errors, and overall model fit. Given the clear structure of the factors, no rotation was needed in the factor analysis. The significant Bartlett sphericity test and the KMO test with a value of 0.81 confirmed the validity of the structure for CFA.

**Table 2:** Descriptive Distribution of Primary Care Assessment Tool Attributes and Overall Scores in the Brazilian Adults Reduced Version, with Internal Consistency Tested by Cronbach's Alpha.

		Number of Items	Mean of PCATool Scores* (SD)	Cronbach's Alpha
Degree of Affiliation		3	0.82 (2.55)	0.72
PCATool (Overall Scale)		25	5.61 (1.77)	0.79
Attributes				
	First Contact	3	5.31 (2.39)	0.77
	Longitudinality	4	6.75 (2.48)	0.76
	Care Coordination	5	4.68 (2.02)	0.77
	Comprehensiveness	7	5.97 (2.71)	0.77
	Family Orientation	2	4.94 (3.35)	0.76
	Community Orientation	1	4.10 (3.94)	0.78

* Ranges from 0 to 10. PCATool: Primary Care Assessment Tool; SD: standard deviation.

CFA results are presented in Table 3, along with the factor loadings. The PCATool-adults latent variable was associated, through standardized factor loadings, with two specific items of the questionnaire — one related to the Degree of Affiliation attribute and the other to the Community Orientation attribute. Additionally, it was connected to five latent variables comprising 21 items, representing the remaining five attributes of the PCATool-adults. The final global adjustments of the model were: CFI=0.980; TLI=0.974; RMSEA=0.007 (90%CI 0.007–0.007); and SRMR=0.062. In addition, specific correlations were used to show the better model, as detailed in Table 4. These correlations were examined to assess the relationships between various PCATool-adults attributes and items. The internal consistency of the model tested by Cronbach's Alpha is shown in Table 2 and was 0.79. The Cronbach's Alpha for the seven factors was also higher than 0.70, demonstrating a good correlation between the different items within the structure.

**Table 3:** Standardized estimated effects of indicators in initial and final dimensional models of the reduced version of the Primary Care Assessment Tool by confirmatory factor analysis. Brazilian National Health Survey, 2019.

		Factor loadings*	
		Initial model	Final model†
PCATool			
	Degree of Affiliation (A1+A2+A3)	0.85 (p<0.00)	0.60 (p<0.00)
	First Contanct	0.96 (p<0.00)	0.53 (p<0.00)
	Longitudinality	0.74 (p<0.00)	0.94 (p<0.00)
	Care Coordination	0.52 (p<0.00)	0.77 (p<0.00)
	Comprehensiveness	0.39 (p<0.00)	0.48 (p<0.00)
	Family Orientation	0.56 (p<0.00)	0.69 (p<0.00)
	Community Orientation (J4)	0.26 (p<0.00)	0.35 (p<0.00)
First Contact			
	B2	0.76 (p<0.00)	0.58 (p<0.00)
	C4	0.24 (p<0.00)	0.28 (p<0.00)
	C11	0.12 (p<0.00)	0.14 (p<0.00)
Longitudinality			
	D1	0.56 (p<0.00)	0.55 (p<0.00)
	D6	0.66 (p<0.00)	0.54 (p<0.00)
	D9	0.81 (p<0.00)	0.73 (p<0.00)
	D14	0.32 (p<0.00)	0.34 (p<0.00)
Care Coordination			
	E2	0.83 (p<0.00)	0.77 (p<0.00)
	E6	0.77 (p<0.00)	0.70 (p<0.00)
	E7	0.82 (p<0.00)	0.82 (p<0.00)
	E9	0.81 (p<0.00)	0.82 (p<0.00)
	F3	0.05 (p<0.00)	0.06 (p<0.00)
Comprehensiveness			
	G9	0.59 (p<0.00)	0.50 (p<0.00)
	G17	0.56 (p<0.00)	0.46 (p<0.00)
	G20	0.68 (p<0.00)	0.58 (p<0.00)
	H1	0.73 (p<0.00)	0.70 (p<0.00)
	H5	0.72 (p<0.00)	0.68 (p<0.00)
	H7	0.62 (p<0.00)	0.67 (p<0.00)
	H11	0.69 (p<0.00)	0.70 (p<0.00)
Family Orientation			
	I1	0.67 (p<0.00)	0.72 (p<0.00)
	I3	0.77 (p<0.00)	0.70 (p<0.00)
Model Fit			
	RMSEA (90%CI)	0.021 (0.021–0.021)	0.007 (0.007–0.007)
	CFI	0.789	0.980
	TLI	0.764	0.974
	SRMR	0.115	0.062

* Taking into account the sample weight;

† The difference between the initial and final models in factor loading analysis lies in the adjustment of the variables, where the final model refines the associations between factors and items, resulting in a more accurate and representative structure of the data. PCATool: Primary Care Assessment Tool; RMSEA: Root Mean Square Error of Approximation; CI: Confidence interval; CFI: Comparative Fit Index; TLI: Tucker-Lewis Index; SRMR: Standardized Root Mean Square Residual.

**Table 4:** Factor Correlation of Dimensional Models in the Reduced Version of the Primary Care Assessment Tool Analyzed via Confirmatory Factor Analysis. Brazilian National Health Survey, 2019.

Factors Correlation	Standardized Coefficients*
First Contact ↔ Care Coordination	-0.896†
Longitudinality ↔ Care Coordination	-0.664†
Comprehensiveness ↔ Care Coordination	-0.291† *
Family Orientation ↔ Care Coordination	-0.567†
Comprehensiveness ↔ Family Orientation	0.525†
Degree of Affiliation (A1+A2+A3) ↔ B2	0.281†
Degree of Affiliation (A1+A2+A3) ↔ D1	0.185†
Degree of Affiliation (A1+A2+A3) ↔ E2	0.136†
Community Orientation (J4) ↔ E2	-0.161†
Community Orientation (J4) ↔ E6	-0.150†
Community Orientation (J4) ↔ E7	-0.203†
Community Orientation (J4) ↔ E9	-0.186†
E6 ↔ E2	0.463†
E6 ↔ D6	-0.037†
E6 ↔ C4	-0.022†
E6 ↔ D9	-0.027†
H1 ↔ H5	0.348†
D6 ↔ D9	0.354†
E2 ↔ D1	0.070†
E2 ↔ B2	0.071†
E2 ↔ D9	0.023†
D14 ↔ C11	0.302†
G20 ↔ G9	0.408†
G20 ↔ G17	0.341†
G9 ↔ G17	0.373†
I3 ↔ E6	-0.033†
I1 ↔ E6	-0.023†
E7 ↔ E9	0.272†
B2 ↔ D1	0.260†
G20 ↔ H11	0.181†

* Taking into account the sample weight;

† p<0.05.

## DISCUSSION

The objective of this study was to evaluate the psychometric properties of the reduced version of the PCATool for adult patients using data from the 2019 National Health Survey, a population-based study with national representation. The results of the exploratory and confirmatory factor analysis carried out in this study confirmed the attributes of primary care originally proposed by Shi and Starfield during the development of the PCATool-Adults^14^. These findings provide a significant contribution to the advancement of knowledge by utilizing a robust, nationally representative sample, which is essential in psychometric analysis. Additionally, the study conducted confirmatory factor analysis, enabling the verification of factor loadings for each component of the PCATool instrument within the Brazilian context.

Prior research on the psychometric properties of PCATool-Adults used exploratory factor analysis only^8^. While this data driven approach is relevant for the identification of patterns in a set of questions^15^, exploratory factor analysis makes no *a priori* specifications in relation to the number of common factors in a measurement scale, which is essential in the case of theory-based scales such as PCATool-Adults. Additional problems of exploratory factor analysis include split loading, when variables load onto more than one factor, and when variables correlate with each other to produce a factor despite having little to no underlying meaning for it^15^.

The present study went further and used exploratory factor analysis and confirmed the adequacy of the model's fit to the data. As expected, the seven attributes of Primary Care originally proposed by Shi and Starfield^14^ were identified, aligning with those of the study that initially developed the reduction of PCATool-Adults^8,14^.

CFA adds to exploratory factor analysis by using a theoretically supported model, which is crucial for a scale like PCATool-Adults, designed to measure PHC attributes that are heterogeneous in nature. This study employed CFA based on this premise, marking a significant update from prior analyses conducted more than a decade ago^8^.

While the findings of the analysis presented in this study were obtained using a representative sample of the Brazilian population, they should be interpreted with caution. Two items, C11 and F3, presented factor loadings of 0.14 and 0.06, respectively, which are far below the threshold of 0.3 that has been recommended for deletion of items in confirmatory factor analysis^16^. In future updates of the questionnaire, this question may be reworded to better capture this attribute. Another limitation of CFA is that its output does not inform whether an item with a low primary loading factor, as in the case of C11 and F3, should have been allotted to another factor or if it should have been interpreted as cross-loading^17–19^. Also, the high correlation observed between different PCATool attributes might suggest some overlapping of these theoretical constructs^13,17,18^.

Overall, the exploratory and confirmatory factor analysis showed good model fit, suggesting that the reduced version of PCATool-Adults captures the attributes of PHC originally devised by Shi and Starfield,^14^ in a sample representative of the Brazilian population. The results reported here are supportive of the use of the short version of PCATool-Adults in health surveys due to its ability to capture PHC attributes and ease of use, when compared to the full version of the scale. The potential of this reduced version includes being less time-consuming, which is an advantage for both researchers and survey participants.

## Supplementary Material



## References

[1] Starfield B, Shi L, Macinko J (2005). Contribution of primary care to health systems and health. Milbank Q.

[2] Mendes EV (2012). O cuidado das condições crônicas na atenção primária à saúde: o imperativo da consolidação da estratégia da saúde da família.

[3] Brasil (2020). Ministério da Saúde. Secretaria de Atenção Primária à Saúde. Departamento de Saúde da Família. Manual do instrumento de avaliação da atenção primária à saúde: PCATool-Brasil: 2020.

[4] Harzheim E, Duncan BB, Stein AT, Cunha CRH, Goncalves MR, Trindade TG (2006). Quality and effectiveness of different approaches to primary care delivery in Brazil. BMC Health Serv Res.

[5] Donabedian A (1978). The quality of medical care. Science.

[6] Shi L, Starfield B, Xu J (2001). Validating the adult primary care assessment tool. J Fam Pract.

[7] Harzheim E, Oliveira MMC, Agostinho MR, Hauser L, Stein AT, Gonçalves MR (2013). Validação do instrumento de avaliação da atenção primária à saúde: PCATool-Brasil adultos. Rev Bras Med Fam Comunidade.

[8] Oliveira MMC, Harzheim E, Riboldi J, Duncan BB (2013). PCATool-ADULTO-BRASIL: uma versão reduzida. Rev Bras Med Fam Comunidade.

[9] Stopa SR, Szwarcwald CL, Oliveira MM, Gouvea ECDP, Vieira MLFP, Freitas MPS (2020). Pesquisa Nacional de Saúde 2019: histórico, métodos e perspectivas. Epidemiol Serv Saude.

[10] Tabachnick BG, Fidell LS (1996). Using multivariate statistics.

[11] Cautin RL, Lilienfeld SO (2015). The encyclopedia of clinical psychology.

[12] Hooper D, Coughlan J, Mullen M (2007). Structural equation modeling: guidelines for determining model fit. Electronic Journal of Business Research Methods.

[13] Martins GA (2006). Sobre confiabilidade e validade. RBGN.

[14] Shi L, Starfield B (2000). Primary care, income inequality, and self-rated health in the United States: a mixed-level analysis. Int J Health Serv.

[15] Yong AG, Pearce S (2013). A Beginner's guide to factor analysis: focusing on exploratory factor analysis. Tutor Quant Methods Psychol.

[16] Hair JF, Tatham RL, Anderson RE, Black W (1998). Multivariate data analysis.

[17] Prudon P (2015). Confirmatory factor analysis as a tool in research using questionnaires: a critique. Comprehensive Psychology.

[18] Hooper D, Coughlan J, Mullen M (2008). Structural equation modeling: guidelines for determining model fit. Electronic Journal of Business Research Methods.

