# Supplementary material for: Psychometric properties of the reduced version of the Primary Care Assessment Tool (PCATool)
Source: Rev Bras Epidemiol. 2024 Dec 9;27:e240057. doi: 10.1590/1980-549720240057 (PMC11656497; doi:10.1590/1980-549720240057)
Supplement: Supplementary file 1 [file 1980-5497-rbepid-27-e240057-suppl1.docx]

**Supplementary material**

| **Supplementary Table 1 – Factorial loading matrix for the multimorbidity patterns derived with ten chronic conditions plus Class III obesity (≥40kg/m²) found in women aged 20-69 years, São Leopoldo, RS, Brazil, 2015 (n=1128).** | | | | |
| --- | --- | --- | --- | --- |
|  | **Factor/Pattern** | | | |
|  | **Cardiometabolic** | **Endocrine-articular** | **Psychosomatic** | **Morbid Obesity** |
| Dyslipidemia | **0.7415** | 0.1843 | -0.0624 | 0.0080 |
| Circulatory disorders | **0.6724** | -0.1295 | 0.0202 | -0.0199 |
| Arterial hypertension | **0.6353** | -0.0554 | 0.0499 | 0.0026 |
| Diabetes | **0.6308** | 0.1487 | 0.1663 | 0.3418 |
| Thyroid diseases | **0.4219** | 0.2209 | **0.4004** | -0.3051 |
| Osteoporosis/Osteopenia | -0.0753 | **0.7026** | 0.0201 | 0.0193 |
| Rheumatic diseases | 0.0757 | **0.6195** | -0.0350 | -0.0131 |
| Chronic pain | 0.0438 | **0.5958** | 0.1162 | -0.0017 |
| Commom mental disorders (CMD) | 0.0081 | 0.0286 | **0.7053** | 0.0251 |
| Acid-related digestive disorders | 0.0439 | 0.0232 | **0.7042** | 0.0575 |
| Morbid Obesity^b^ | 0.0809 | 0.0061 | 0.0578 | **0.9260** |
| ***Explained variance %^c^*** | ***20.2*** | ***12.1*** | ***9.6*** | ***9.4*** |
| ^a^Factor loadings indicate the strength of association between each variable and each fator, with a factor loading of <0.3 (non-bold loadings) generally considered to be weak; ^b^BMI≥40Kg/m²(WHO CONSULTATION ON OBESITY (1999: GENEVA; ORGANIZATION. 2000);  ^c^The percentage of total variance accounted by all factors is 51.3%. | | | | |

| **Supplementary Table 2 – Crude and adjusted sleep medication use analyses according to low and high scores of multimorbidity patterns derived with ten chronic conditions in the different adjustment models in women in Southern Brazil (n=1128).** | | | | |
| --- | --- | --- | --- | --- |
|  | **Model I** | **Model II** | **Model III** | **Model IV** |
|  | **PR (CI95%)** | **PR (CI95%)** | **PR (CI95%)** | **PR (CI95%)** |
| **Cardiometabolic** |  |  |  |  |
| p-value* | **<0,001** | **0,008** | 0,062 | 0,163 |
| Low | 1 | 1 | 1 | 1 |
| High | 2,36 (1,78-3,12) | 1,58 (1,13-2,21) | 1,41 (0,98-2,02) | 1,27 (0,91-1,79) |
| **Endocrine-articular** |  |  |  |  |
| p-value* | **0,001** | 0,401 | 0,537 | 0,499 |
| Low | 1 | 1 | 1 | 1 |
| High | 1,63 (1,21-2,18) | 1,15 (0,83-1,59) | 1,12 (0,78-1,60) | 1,12 (0,80-1,57) |
| **Psychosomatic** |  |  |  |  |
| p-value* | **<0,001** | **<0,001** | **<0,001** | **<0,001** |
| Low | 1 | 1 | 1 | 1 |
| High | 4,51 (3,34-6,11) | 3,57 (2,57-4,97) | 3,44 (2,41-4,90) | 2,81 (1,99-3,96) |
| PR: Prevalence Ratio; CI95%: confidence level of 95%; Model I: unadjused prevalence ratio; Model II: Model I + sociodemographic variables; Model III: Model II + behavioral variable; Model IV: Model III + health variables. *Variables associated with use of sleep medications with a p-value ≤0.20 were kept in the model as potential confounding factors. | | | | |

| **Supplementary Table 3 - Prevalence of high scores for multimorbidity patterns according to sociodemographic, behavioral and health characteristics in women in Southern Brazil (n=1128).** | | | | | | | | |
| --- | --- | --- | --- | --- | --- | --- | --- | --- |
| **Variables** | **Multimorbidity Patterns** | | | | | | | |
|  | **Cardiometabolic** | | **Endocrine-articular** | | **Psychosomatic** | | **Morbid Obesity***** | |
|  | **n (%)** | **p-value** | **n (%)** | **p-value** | **n (%)** | **p-value** | **n (%)** | **p-value** |
| **Prevalence** | 343 (30.6) |  | 315 (28.1) |  | 363 (32.4) |  | 354 (31.6) |  |
| **Age (years)** |  | **<0.001^b^** |  | **<0.001^b^** |  | **<0.001^b^** |  | **<0.001^b^** |
| 20-44 | 72 (11.9) |  | 94 (15.5) |  | 130 (21.5) |  | 124 (20.5) |  |
| 45-69 | 271 (52.4) |  | 221 (42.8) |  | 233 (45.1) |  | 230 (44.5) |  |
| **Skin color** |  | **0.055^b^** |  | **0.046^b^** |  | **0.481^b^** |  | **0.543^b^** |
| White | 242 (29.0) |  | 221 (26.5) |  | 265 (31.8) |  | 259 (31.1) |  |
| Other (Black, brown, indigenous or yellow) | 101 (35.1) |  | 94 (32.6) |  | 98 (34.0) |  | 95 (33.0) |  |
| **Marital status** |  | **0.317^b^** |  | **0.253^b^** |  | **0.249^b^** |  | **0.747^b^** |
| Not having a partner | 117 (28.8) |  | 106 (26.0) |  | 240 (33.6) |  | 126 (31.0) |  |
| Having a partner | 226 (31.6) |  | 209 (29.2) |  | 123 (30.2) |  | 228 (31.9) |  |
| **Education (years)** |  | **<0.001^a^** |  | **<0.001^a^** |  | **<0.001^a^** |  | **<0.001^a^** |
| ≥11 | 88 (18.9) |  | 97 (20.8) |  | 108 (23.2) |  | 105 (22.5) |  |
| 8 – 10 | 50 (25.1) |  | 51 (25.6) |  | 54 (27.1) |  | 50 (25.1) |  |
| 5 – 7 | 100 (39.7) |  | 91 (36.1) |  | 113 (44.8) |  | 113 (44.8) |  |
| 0 – 4 | 103 (50.7) |  | 75 (37.0) |  | 88 (43.6) |  | 84 (41.4) |  |
| **Economy class (ABEP)** |  | **0.022^a^** |  | **0.478^a^** |  | **0.237^a^** |  | **0.785^a^** |
| A + B | 96 (24.7) |  | 97 (25.0) |  | 111 (28.6) |  | 116 (29.9) |  |
| C | 202 (34.1) |  | 184 (31.1) |  | 211 (35.7) |  | 201 (34.0) |  |
| D + E | 42 (30.9) |  | 33 (24.3) |  | 41 (30.2) |  | 34 (25.0) |  |
| **Household income per capita (quartile)** |  | **0.765^a^** |  | **0.030^a^** |  | **0.658^a^** |  | **0.444^a^** |
| I (low) | 76 (27.9) |  | 65 (23.9) |  | 80 (29.4) |  | 79 (29.0) |  |
| II | 82 (30.2) |  | 66 (24.3) |  | 89 (32.7) |  | 88 (32.6) |  |
| III | 99 (36.5) |  | 94 (34.7) |  | 99 (36.5) |  | 91 (33.6) |  |
| IV (high) | 73 (27.0) |  | 79 (29.3) |  | 81 (30.0) |  | 86 (31.9) |  |
| **Occupation status** |  | **<0.001^b^** |  | **<0.001^b^** |  | **<0.001^b^** |  | **<0.001^b^** |
| Employed | 128 (19.7) |  | 141 (21.7) |  | 169 (26.0) |  | 158 (24.3) |  |
| Unemployed | 215 (45.7) |  | 174 (37.0) |  | 193 (41.1) |  | 195 (41.5) |  |
| **Consumption of fruits and vegetables** |  | **<0.001^b^** |  | **<0.001^b^** |  | **0.002^b^** |  | **<0.001^b^** |
| Adequate (≥5 servings daily) | 194 (39.7) |  | 175 (35.8) |  | 182 (37.2) |  | 183 (37.4) |  |
| Inadequate (<5 servings daily) | 149 (23.6) |  | 140 (22.2) |  | 181 (28.7) |  | 171 (27.1) |  |
| **Alcohol consumption** |  | **<0.001^b^** |  | **<0.001^b^** |  | **<0.001^b^** |  | **<0.001^b^** |
| Does not consume | 147 (42.7) |  | 121 (35.2) |  | 146 (42.4) |  | 135 (39.2) |  |
| Consumes | 153 (22.1) |  | 164 (23.6) |  | 183 (26.4) |  | 187 (27.0) |  |
| **Smoking status** |  | **0.001^b^** |  | **<0.001^b^** |  | **0.023^b^** |  | **0.221^b^** |
| Non-smoker | 185 (28.2) |  | 172 (26.2) |  | 194 (29.5) |  | 194 (29.5) |  |
| Former smoker | 103 (40.1) |  | 96 (37.4) |  | 100 (38.9) |  | 89 (34.6) |  |
| Current smoker | 55 (26.4) |  | 47 (22.6) |  | 69 (33.2) |  | 71 (34.1) |  |
| **Physical activity** |  | **0.250^b^** |  | **0.733^b^** |  | **0.354^b^** |  | **0.487^b^** |
| Active | 43 (26.7) |  | 47 (29.2) |  | 47 (29.2) |  | 47 (29.2) |  |
| Inactive | 300 (31.2) |  | 268 (27.9) |  | 316 (32.9) |  | 307 (32.0) |  |
| **Body mass index*** |  | **<0.001^a^** |  | **<0.001^a^** |  | **<0.001^a^** |  | **<0.001^a^** |
| Low weight/Eutrophy | 50 (13.2) |  | 52 (13.7) |  | 72 (19.0) |  | 72 (19.0) |  |
| Overweight | 112 (30.0) |  | 99 (26.5) |  | 116 (31.1) |  | 101 (27.1) |  |
| Obesity | 181 (49.1) |  | 164 (44.4) |  | 175 (47.4) |  | 181 (49.1) |  |
| **Common mental disorders**** |  | **<0.001^b^** |  | **0.290^b^** |  | **<0.001^b^** |  | **0.005^b^** |
| Absence | 179 (26.6) |  | 181 (26.9) |  | 167 (24.8) |  | 191 (28.3) |  |
| Presence | 163 (36.5) |  | 133 (29.8) |  | 195 (43.7) |  | 162 (36.2) |  |
| **Use of sleeping pills** |  | **<0.001^b^** |  | **0.002^b^** |  | **<0.001^b^** |  | **<0.001^b^** |
| No | 260 (27.0) |  | 254 (26.4) |  | 255 (26.5) |  | 271 (28.2) |  |
| Yes | 83 (51.9) |  | 61 (38.1) |  | 108 (67.5) |  | 83 (51.9) |  |
| *Body mass index (BMI): Low Weight/Eutrophy <25 Kg/m², Overweight 25 to 29.9 Kg/m², Obesity ≥30 Kg/m²; **Common mental disorders (CMD) = score SRQ ≥7; ***Morbid obesity = BMI ≥ 40 kg/m²; ^a^p-value of the chi-square test for linear trend; ^b^p-value of the chi-square test for heterogeneity of proportions. | | | | | | | | |

| **Supplementary Table 4 - Crude and adjusted sleep medication use analyses according to low and high score of multimorbidity patterns derived with ten chronic conditions plus Class III obesity (≥40kg/m²) in the different adjustment models in women in Southern Brazil (n=1128).** | | | | |
| --- | --- | --- | --- | --- |
|  | **Model I** | **Model II** | **Model III** | **Model IV** |
|  | **PR (CI95%)** | **PR (CI95%)** | **PR (CI95%)** | **PR (CI95%)** |
| **Cardiometabolic** |  |  |  |  |
| p-value* | **<0,001** | **0,003** | **0,039** | 0,118 |
| Low | **1** | **1** | **1** | **1** |
| High | 2,45 (1,84-3,25) | 1,66 (1,19-2,32) | 1,46 (1,02-2,09) | 1,31 (0,93-1,85) |
| **Endocrine-Articular** |  |  |  |  |
| p-value* | **0,002** | 0,388 | 0,555 | 0,597 |
| Low | **1** | **1** | **1** | **1** |
| High | 1,58 (1,18-2,11) | 1,15 (0,84-1,58) | 1,11 (0,78-1,57) | 1,09 (0,79-1,51) |
| **Psychosomatic** |  |  |  |  |
| p-value* | **<0,001** | **<0,001** | **<0,001** | **<0,001** |
| Low | **1** | **1** | **1** | **1** |
| High | 4,34 (3,20-5,90) | 3,56 (2,58-4,92) | 3,39 (2,39-4,79) | 2,76 (1,97-3,87) |
| **Morbid Obesity** |  |  |  |  |
| p-value* | **<0,001** | **<0,001** | **<0,001** | **0,001** |
| Low | **1** | **1** | **1** | **1** |
| High | 2,34 (1,76-3,10) | 1,76 (1,29-2,38) | 1,83 (1,32-2,53) | 1,68 (1,24-2,27) |
| PR: Prevalence Ratio; CI95%: confidence level of 95%; Model I: unadjused prevalence ratio; Model II: Model I + sociodemographic variables; Model III: Model II + behavioral variable; Model IV: Model III + health variables; *Variables associated with use of sleep medications with a p-value ≤0.20 were kept in the model as potential confounding factors. | | | | |

**Supplementary Table 5 –** **Crude and adjusted sleep medication use analyses according to low and high score of multimorbidity patterns derived with ten chronic conditions plus Class III obesity (≥40kg/m²) in the different adjustment models according to the age groups <45 years and ≥45 years in women in Southern Brazil (n=1128).**

|  | **Model I** | | **Model II** | | **Model III** | | **Model IV** | |
| --- | --- | --- | --- | --- | --- | --- | --- | --- |
|  | **<45** | **≥45** | **<45** | **≥45** | **<45** | **≥45** | **<45** | **≥45** |
|  | **PR (CI95%)** | **PR (CI95%)** | **PR (CI95%)** | **PR (CI95%)** | **PR (CI95%)** | **PR (CI95%)** | **PR (CI95%)** | **PR (CI95%)** |
| **Cardiometabolic** | | | | | | | | |
| p-value* | **0.002** | **0.005** | **0.001** | **0.031** | **0.002** | 0.183 | **0.017** | 0.292 |
| Low | 1 | 1 | 1 | 1 | 1 | 1 | 1 | 1 |
| High | 2.47  (1.38-4.40) | 1.67  (1.17-2.39) | 2.47  (1.42-4.32) | 1.51  (1.04-2.19) | 2.43  (1.40-4.23) | 1.31  (0.88-1.94) | 1.90  (1.12-3.21) | 1.23  (0.83-1.82) |
| **Endocrine-articular** | | | | | | | | |
| p-value* | **0.006** | 0.971 | **0.004** | 0.675 | **0.006** | 0.554 | **0.037** | 0.665 |
| Low | 1 | 1 | 1 | 1 | 1 | 1 | 1 | 1 |
| High | 2.20  (1.26-3.85) | 0.99  (0.71-1.40) | 2.26  (1.30-3.94) | 0.93  (0.66-1.31) | 2.19  (1.25-3.84) | 0.89  (0.61-1.30) | 1.79  (1.04-3.09) | 0.92  (0.65-1.32) |
| **Psychosomatic** | | | | | | | | |
| p-value* | **<0.001** | **<0.001** | **<0.001** | **<0.001** | **<0.001** | **<0.001** | **<0.001** | **0.001** |
| Low | 1 | 1 | 1 | 1 | 1 | 1 | 1 | 1 |
| High | 5.85  (3.46-9.88) | 2.89  (1.99-4.21) | 5.38  (3.23-8.96) | 2.68  (1.85-3.90) | 5.27  (3.18-8.76) | 2.46  (1.63-3.70) | 4.31  (2.60-7.14) | 2.03  (1.35-3.05) |
| **Morbid Obesity** | | | | | | | | |
| p-value* | **<0.001** | **0.018** | **<0.001** | 0.066 | **<0.001** | 0.088 | **<0.001** | 0.099 |
| Low | **1** | **1** | **1** | **1** | **1** | **1** | **1** | **1** |
| High | 3.32  (2.00-5.53) | 1.50  (1.07-2.10) | 3.01  (1.82-4.97) | 1.38  (0.98-1.94) | 3.03  (1.84-5.00) | 1.38  (0.95-2.01) | 2.59  (1.56-4.29) | 1.34  (0.94-1.90) |

PR: Prevalence Ratio; CI95%: confidence level of 95%; Model I: unadjused prevalence ratio; Model II: Model I + sociodemographic variables; Model III: Model II + behavioral variable; Model IV: Model III + health variables; *Variables associated with use of sleep medications with a p-value ≤0.20 were kept in the model as potential confounding factors.
